# Occurrence of Selected Zoonotic Fecal Pathogens and First Molecular Identification of* Hafnia paralvei* in Wild Taihangshan Macaques (*Macaca mulatta tcheliensis*) in China

**DOI:** 10.1155/2019/2494913

**Published:** 2019-04-03

**Authors:** Qingxun Zhang, Shuyi Han, Kongshang Liu, Jing Luo, Jiqi Lu, Hongxuan He

**Affiliations:** ^1^National Research Center for Wildlife-Borne Diseases, Institute of Zoology, Chinese Academy of Sciences, Beijing, China; ^2^University of the Chinese Academy of Sciences, Beijing, China; ^3^Institute of Biodiversity and Ecology, Zhengzhou University, Zhengzhou, China

## Abstract

Rhesus macaques (*Macaca mulatta*) are hosts to a range of zoonotic and potentially zoonotic pathogens. The present study firstly provides a broader investigation of the presence and prevalence of zoonotic fecal pathogens in wild Taihangshan macaques, a subspecies of rhesus macaque in China. A total of 458 fecal samples were collected between September 2015 and November 2016. Fourteen genera of intestinal parasites (four genera of protozoans and ten genera of helminths) and twelve genera of bacteria were tested for using PCR amplification. The overall samples prevalence of parasitic infection was 98.25%.* Entamoeba *spp. (89.96%),* Balantidium coli* (70.09%), and* Isospora* spp. (28.38%) were the most prevalent protozoa, whereas the predominant prevalent helminths were* Trichuris* sp. (93.23%),* Strongyloides *spp. (73.36%), and* Oesophagostomum* sp. (31.66%). Ten genera of intestinal bacteria were detected in samples of rhesus macaques, including* Shigella* (31.66%),* Escherichia coli* (29.91%),* Klebsiella pneumoniae* (28.38%),* Leptospira* (26.64%),* Campylobacter jejuni *(18.34%),* Salmonella *(13.32%), etc. Eight samples (1.75%) were tested* Hafnia*-positive based on sequences analysis of 16S rRNA and ampC gene. This is the first molecular characterization of* Hafnia* infection in NHPs. Our cross-sectional prevalence study provides important information for monitoring the potential transmission of zoonotic infections from wild rhesus macaques.

## 1. Introduction

Rhesus macaques (*Macaca mulatta*) are one of the most widely distributed nonhuman primates (NHPs) in the world. There are more than six kinds of rhesus macaque subspecies in China, and Taihangshan macaque (*Macaca mulatta tcheliensis*) is a subspecies confined to China southern Taihangshan Mountains area on the Henan-Shanxi border (N 35°12′49′′, E 112°41′25′′) [1]. Rhesus macaques are an increasingly important source of zoonotic diseases [2]. They can share pathogens with livestock and humans and act as reservoirs for several emerging infectious diseases such as malaria [2, 3]. Some molecular epidemiological studies have been performed to evaluate the prevalence of bacteria and parasites in NHPs [4–10]. Enterobacterial pathogens including* Shigella* spp.,* Salmonella* spp., and* Escherichia coli* are the most commonly distributed pathogens in NHPs [4–6]. A diversity of intestinal parasites including five protozoan genera and six helminths genera was reported to infect NHPs [7, 8]. Many of these bacteria and parasites have strong pathogenic potential in both humans and animals. More recently, investigations of parasitic infections have been reported in several subspecies of rhesus macaque samples in China [7, 9, 10]. However, there are few data regarding the prevalence and distribution of the potential zoonotic fecal pathogens among Taihangshan macaques. Therefore, our objective was to get initial information about the presence and prevalence of selected fecal pathogens in wild Taihangshan macaques.

## 2. Material and Methods

### 2.1. Sample Collection

During September 2015 to November 2016, a total of 458 fecal samples (9 liquid stools from nine different individuals) were collected from wild Taihangshan macaques in Wulongkou Scenic Area (N 35°12′49′′, E 112°41′25′′), Jiyuan, Henan Province of China. Fresh droppings were collected from each animal using individual sterile cryogenic vials and immediately placed in a cooler with ice packs and transported to the laboratory.

### 2.2. Sample Processing and PCR Analysis

Genomic DNA was extracted from 200 mg of each fecal sample using the QIAamp® DNA Stool Mini Kit (QIAGEN, Hilden, Germany) according to the manufacturer's instructions. Extracted nucleic acids were frozen at −20°C prior to the further study. To explore the molecular epidemiology of zoonotic pathogens in Taihangshan macaques, fourteen genera of intestinal parasites (four genera of protozoans and ten genera of helminths) and thirteen genera of bacteria were tested for using PCR amplification.* Hafnia* was amplified with conventional PCR using the primers specific for the 16S rRNA gene and ampC gene as described previously [11, 12]. All primers and conditions used in this study were listed in Supplementary Table 1.

### 2.3. Isolation, Genotyping, and Antibacterial Susceptibility of the* Hafnia*

The fecal samples were cultured on nutrient agar plates (Oxoid, UK) in anaerobic conditions at 37°C for 18 h and typical colonies were transferred at least thrice in the same conditions. The isolates were examined by the Gram stain, 16S rRNA gene and ampC gene sequencing, and biochemical tests using the BD Phoenix Automated Microbiology System (BD). Antibiotic susceptibility testing was performed for* Hafnia* as previously described [12].

### 2.4. Sequencing and Phylogenetic Analyses

The PCR products from positive samples were bidirectionally sequenced at BGI Sequencing (Beijing, China). Nucleotide sequences were aligned with reference strains obtained from the GenBank database. The molecular phylogenetic trees were constructed by using the neighbor-joining method executed in MEGA6 [13]. The robustness of the tree topology was assessed with 1000 bootstrap replicates.

### 2.5. Nucleotide Sequence Accession Numbers

The representative nucleotide sequences of this study have been deposited in the GenBank database under accession number MG923797 for 16S rRNA gene and MK189458 for ampC gene.

## 3. Results

### 3.1. Prevalence of Parasitic Pathogens in Wild Taihangshan Macaques Samples

Overall, 450 (98.25%) of the 458 Taihangshan macaques fecal samples were infected with one or more parasites. The majority of gastrointestinal parasites found in the Taihangshan macaques exhibit relatively high samples prevalence.* Entamoeba* spp. was the most prevalent protozoa and its prevalence was 89.96%, followed by 70.09% prevalence of* Balantidium coli*, and 28.38% of* Isospora* spp., whereas the predominant prevalent helminths were* Trichuris* sp. (93.23%),* Strongyloides *spp. (73.36%),* Oesophagostomum* sp. (31.66%),* Physaloptera* sp. (15.07%),* Capillaria* spp. (8.30%),* Trichostrongylus* sp. (6.77%),* Ancylostoma *spp. (3.28%), and* Enterobius *spp. (1.75%) (Table 1). All samples were negative for* Ascaris* sp.,* Cryptosporidium* spp., and* Gongylonema pulchrum*.

### 3.2. Prevalence of Bacterial Pathogens in Wild Taihangshan Macaques Samples

Ten genera of intestinal bacteria tested for were found in the Taihangshan macaques. The overall prevalence of selected bacterial infection was 84.93% (389/458). More in detail, 61 cases tested positive for* Salmonella* (infection rate 13.32%), 145 for* Shigella* (31.66%), 137 for* Escherichia coli *(29.91%), 130 for* Klebsiella pneumoniae* (28.38%), 31 for* Yersinia* (6.77%), 84 for* Campylobacter jejuni* (18.34%), 53 for* Staphylococcus aureus* (11.57%), 122 for* Leptospira* (26.64%), and 38 for* Streptococcus pneumoniae* (8.30%) (Table 1). Among these species,* Shigella* was the most frequently detected bacteria, followed by* Escherichia coli* and* Klebsiella pneumoniae*. Of the 9 liquid stools from nine different monkeys, the positive rate of* Shigella* was 100%. All samples tested negative for* Mycobacterium tuberculosis* and* Pasteurella multocida*.

### 3.3. Molecular Characterization of* Hafnia paralvei* in Wild Taihangshan Macaques Samples

**Figure 1:**
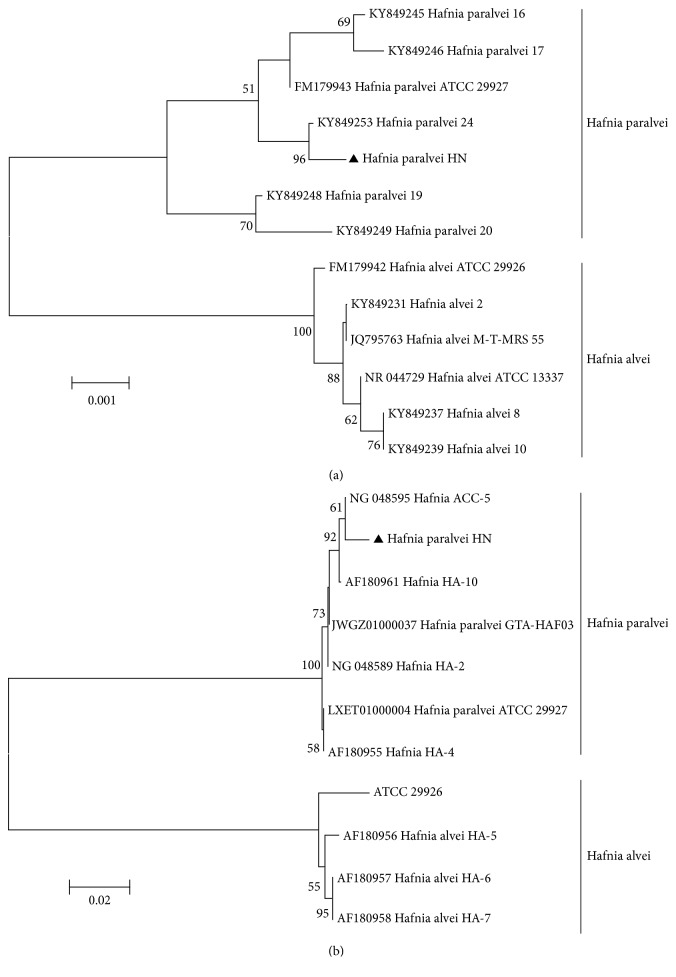
Phylogenetic analyses of* Hafnia* based on sequences of 16S rRNA gene (approximately 1400 nt) (a) and ampC gene (approximately 759nt) (b) using the neighbor-joining (NJ). Bootstrap values > 50% are shown. The genotypes identified in this study are indicated by ▲.

*Hafnia* infections were observed in 8 samples, with the sample prevalence as 1.75% (8/458). To our knowledge, this is the first genetic characterization of* Hafnia paralvei *infection in NHPs. Sequence analysis revealed that these sequences had similarities of 99.7%-100%. The* H. paralvei HN* strain was successfully isolated from fecal samples. Biochemical studies showed that the isolated* H. paralvei* was typically malonate and *β*-glucosidase negative, while the opposite pattern is associated with* H. alvei* species [12]. The results of the antibiotic analysis of* H. paralvei HN* against 21 different antibacterial agents are demonstrated in Supplementary Table 2. The* H. paralvei HN* strain was sensitive to 14 antibiotic agents especially amikacin, gentamicin, imipenem, meropenem, ceftazidime, cefotaxime, cefepime, aztreonam, piperacillin, piperacillin-tazobactam, trimethoprim-sulfamethoxazole, chloramphenicol, ciprofloxacin, and levofloxacin and resistant to 5 antibiotic agents including cefazolin, ampicillin, amoxicillin-clavulanate, ampicillin-sulbactam, and tetracycline. The type or reference strains used in these studies included two* H. alvei* strains (ATCC 13337 and ATCC 29926) and one* H. paralvei* strain (ATCC 29927). The subsequent molecular phylogeny analysis based on 16S rRNA gene (approximately 1400 nt) revealed that* H. paralvei HN* strain belonged to genotype* H. paralvei*. The strain was most closely related to* H. paralvei *strain 24 (KY849253) isolated from the human rectal swab and share 99% homology (Figure 1). Meanwhile, phylogenetic relationships based on partial* ampC *sequence had remarkably similar results. The strain* H. paralvei HN* shared the highest similarity homology 99% with* Hafnia* ACC-5 (NG 048595) (Figure 1).

## 4. Discussion

Taihangshan macaque (*Macaca mulatta tcheliensis*) occupies the northern limit of all rhesus macaque natural populations in the world [1, 14]. A national nature reserve in the Taihangshan Mountains area was established by the Chinese government to protect the macaques. In most wildlife populations, infectious diseases are considered as the second leading cause of mortality, right behind predation and malnutrition [15]. Meanwhile, rhesus macaques have the potential to transmit various pathogens to humans and domestic animals. Therefore, monitoring the presence and prevalence of zoonotic infectious agents among this population is important for the general health of humans and animals coming into contact with this population.

This study demonstrates a high samples prevalence (98.25%, 450/458) and diversity (three genera of protozoans and eight genera of helminths) of intestinal parasites in Taihangshan macaques in China. Previous studies revealed that the samples prevalence varied significantly with species, geographic region, feeding habitats, and age [7, 8]. Similar prevalence was reported in bushmeat monkeys samples (92%) in Cameroon [8], while lower prevalence was found in 34 NHP species samples (54.1%) in China [7] and primates samples (54.5%) at a zoo in Malaysia [16].

Several molecular epidemiological studies have demonstrated* Trichuris* sp. and* Entamoeba* spp. were the most frequently detected parasites [8], whereas others reported that* Entamoeba* spp. [7] or* Strongyloides* spp. [17] was the most prevalent. Similarly,* Trichuris* sp.,* Entamoeba* spp., and* Strongyloides* spp. were the most common parasitic pathogens in our study with a high prevalence of 93.23%, 89.96%, and 73.36%, respectively. Several studies reported that* Trichuris* sp. and* Entamoeba* spp. were parasitic with a high potential for transmission to humans and animals because of their simple and direct life cycles [7, 18, 19].

*Balantidium coli* detected in our study are multihost parasite capable of infecting animals and humans.* Balantidium coli *could damage the intestinal mucosal and cause serious diarrhea and dysentery [10]. The prevalence of infection found in the present study (70.09%) with* Balantidium coli* is consistent with rates previously published in bred rhesus monkeys and baboons [10, 20], but higher than cercopithecid monkeys samples [21].* Isospora* spp. infections are very common and have a worldwide distribution [22]. The prevalence of* Isospora* spp. DNA in Taihangshan macaques was quite high (28.38%). On the contrary, the previous study in nonhuman primates samples in China from 2009 to 2015 revealed a relatively lower (1.9%) prevalence rate for this protozoan [7]. Macaques in several regions of China and India have been reported to be infected with* Cryptosporidium* spp. [7, 23, 24], while in this study all macaques samples tested by PCR were negative. The negative results suggested that this protozoan is not an important parasite in rhesus macaques in this region.

Even though a diversity of intestinal helminths parasites was frequently reported to infect NHPs [8, 10], samples frequencies of* Trichuris* sp.,* Strongyloides* spp.,* Physaloptera* sp.,* Capillaria* spp.,* Trichostrongylus* sp.,* Ancylostoma* spp., and* Enterobius *spp. were higher than previous studies [7, 21]. However, a relative lower sample infection rate (31.66%) of* Oesophagostomum* sp. was found in all macaque monkeys than wild chimpanzees (48%) and red colobus samples (41%) [25, 26]. Meanwhile, there was no evidence for exposure to* Ascaris* sp. and* Gongylonema pulchrum* within this wild rhesus macaque population. Evidence had shown that helminths (including* Oesophagostomum *sp.,* Ascaris *sp.,* Physaloptera* sp.,* Ancylostoma* spp., and* Enterobius vermicularis*) were parasitic with a high potential for transmission to humans and animals [7, 18, 19, 26]. Together, the high prevalence and diversity of intestinal parasites suggest potential public health risk from this wild rhesus macaque.

The results from the present study confirm the local circulation of zoonotic bacterias, including* Salmonella*,* Shigella*,* Escherichia coli*,* Campylobacter jejuni*, and* Yersinia*. Infections of these pathogens were spread by the fecal-oral route or contacting with pathogen carriers, often of animal and wildlife origin, as well as consumption of contaminated food and water [27].* Shigella* was the most regular pathogen in Enterobacteriaceae and could easily cause human and other animals infection. The samples prevalence of* Shigella* (31.66%) was higher than that reported by Banish et al. [28]. Nine monkeys with clinical manifestations of diarrhea were all positive for* Shigella* infection which suggested that* Shigella* was an important bacterial cause of diarrhea in this region. Different species of NHPs are known to be potential carriers of* Salmonella*,* Escherichia coli*,* Campylobacter jejuni*,* Klebsiella pneumoniae*, and* Leptospira*. Published estimates suggest that the samples prevalence of* Leptospira *was slightly higher in wild-caught vervet monkeys samples [29], while the prevalence of* Escherichia coli *was lower in wild chimpanzees samples [5]. The similar prevalence of* Campylobacter* spp. and* Klebsiella pneumoniae* has been documented in various investigations [30, 31]. Even though wild Taihangshan macaques were not known to be exposed to* Mycobacterium tuberculosis* and* Pasteurella multocida*, rhesus monkeys are known to be infected with these bacteria. Importantly, wild Taihangshan macaques serving as a reservoir host could facilitate the spread of bacterial infection.

*Hafnia*, a gram-negative bacterium belonging to the Enterobacteriaceae family presently consists of at least two distinct relatedness groups (*Hafnia alvei *and* Hafnia paralvei*) [11, 32].* Hafnia *has a worldwide distribution including a variety of mammals, birds, reptiles, amphibians, fish, and foods [33].* Hafnia* is responsible for infectious diseases in pullets, rats, and horses [34, 35] and has been recovered from human clinical specimens, even though it may be opportunistic human pathogens [12, 36–38]. Gunthard and Pennekamp [36] performed a study on the clinical significance of* Hafnia* isolates from 61 patients. The results indicated that* Hafnia* was isolated from 57 patients (93.4%) with underlying illnesses. More importantly,* Hafnia* was found to be the sole etiologic agent of invasive disease in 3 patients characterized by septicemia or peritonitis. In our previous study,* Hafnia* was detected from sample of liver tissue with hemorrhage of a dead rhesus monkey (unpublished data). The present study firstly indicates the existence of* Hafnia paralvei* in NHPs in China. However, further studies are needed to isolate more strains and characterize the associations between the microbiological findings and clinical data of* Hafnia paralvei* in Taihangshan macaques.

## 5. Conclusion

In conclusion, the present study firstly provides a broader investigation of zoonotic pathogens in wild Taihangshan macaques in China, which detailed the presence and prevalence of bacteria and parasites. Our preliminary results demonstrate high prevalence and diversity of significant zoonotic infections amongst wild Taihangshan macaques. This baseline data provides valuable feedback for monitoring the potential transmission of zoonotic infections from wild rhesus macaques.

## Figures and Tables

**Table 1:** Prevalence of pathogens in 458 fecal samples collected from *Macaca mulatta tcheliensis* in China.

Pathogens	No. of positive samples (% positive)	Pathogens	No. of positive samples (% positive)
*Salmonella*	61 (13.32)	*Entamoeba* spp.	412 (89.96)
*Shigella*	145 (31.66)	*Trichuris *sp.	427 (93.23)
*Escherichia coli*	137 (29.91)	*Ascaris* sp.	—
*Klebsiella pneumoniae*	130 (28.38)	*Isospora *spp.	130 (28.38)
*Yersinia*	31 (6.77)	*Physaloptera* sp.	69 (15.07)
*Campylobacter jejuni*	84 (18.34)	*Enterobius* spp.	8 (1.75)
*Staphylococcus aureus*	53 (11.57)	*Strongyloides *spp.	336 (73.36)
*Leptospira*	122 (26.64)	*Ancylostoma *spp.	15 (3.28)
*Streptococcus pneumoniae*	38 (8.30)	*Balantidium coli*	321 (70.09)
*Mycobacterium tuberculosis*	—^a^	*Capillaria *spp.	38 (8.30)
*Hafnia*	8 (1.75)	*Oesophagostomum *sp.	145 (31.66)
*Pasteurella multocida*	—	*Trichostrongylus *sp.	31 (6.77)
*Gongylonema pulchrum*	—	*Cryptosporidium *spp.	—

—^a^, not detected in this study.

## Data Availability

The epidemiological data used to support the findings of this study are included within the article.

## Supplementary Materials

Supplementary Materials Supplementary Table 1 includes pathogens, primer sequences, and references of PCR amplification. Supplementary Table 2 includes antibiotic susceptibility tests of the* H. paralvei *HN.
