# Supplementary material for: Occurrence of Selected Zoonotic Fecal Pathogens and First Molecular Identification of Hafnia paralvei in Wild Taihangshan Macaques (Macaca mulatta tcheliensis) in China
Source: Biomed Res Int. 2019 Apr 3;2019:2494913. doi: 10.1155/2019/2494913 (PMC6530245; doi:10.1155/2019/2494913)
Supplement: Supplementary Materials — Supplementary Table 1 includes pathogens, primer sequences, and references of PCR amplification. Supplementary Table 2 includes antibiotic susceptibility tests of the H. paralvei HN. [file 2494913.f1.docx]

Supplementary Table 1. Pathogens, primer sequences and references of PCR amplification

| Pathogen | Primer sequence (F/R) | References |
| --- | --- | --- |
| *Salmonella* | 5'-TCGCACCGTCAAAGGAACCGTAAAGC-3' | GB/T 28642-2012^a^ |
|  | 5'-GCATTATCGATCAGTACCAGCCGTCT-3' |  |
| *Shigella* | 5'-GTTCCTTGACCGCCTTTCCGATACCGTC-3' | SN/T1869-2007^a^ |
|  | 5'-GCCGGTCAGCCACCCTCTGAGAGTAC-3' |  |
| *Escherichia coli* | 5'-ATTGCGCTGAAGCCTTTG-3' | SN/T1869-2007^a^ |
|  | 5'-CGAGTACATTGGCATCGTG-3' |  |
| *Klebsiella pneumoniae* | 5'-TGGCCCGCGCCCAGGGTTCGAAA-3' | SN/T2641-2010^a^ |
|  | 5'-GATGTCGTCARCGTTGATGCCGAG-3' |  |
| *Yersinia* | 5'-CCGTTATGACACTGTCGCCT-3' | Feng et al.2016 |
|  | 5'-CTGCCCATAGATGCTGCCAT-3' |  |
| *Campylobacter jejuni* | 5'-GATATGTATGATTTTATCTTGC-3' | SN/T1869-2007^a^ |
|  | 5'-GAATGAAATTTTAGAATGGGG-3' |  |
| *Staphylococcus aureus* | 5'-AAAAAAGCACATAACAAGCG-3' | SN/T1869-2007^a^ |
|  | 5'-GATAAAGAAGAAACCAGCAG-3' |  |
| *Leptospira* | 5'-GACCCGAAGCCTGTCGAG-3' | SN/T3741.1-2013^a^ |
|  | 5'-GCCATGCTTAGTCCCGATTAC-3' |  |
| *Streptococcus pneumoniae* | 5'-ATTTCTGTAACAGCTACCAACGA-3' | Salo et al.1995 |
|  | 5'-GAATTCCCTGTCTTTTCAAAGTC-3' |  |
| *Mycobacterium tuberculosis* | 5'-GGAATTCATGACAGAGCAGCAGTGGAATTTC-3' | Han et al.2008 |
|  | 5'-ATTTGCGGCCGCTGCGAACATCCCAGTGACG-3' |  |
| *Pasteurella multocida* | 5'-GACAACGCCCTCAGCARCACCAGC-3' | SN/T2641-2010^a^ |
|  | 5'-CGCTGGCCCATTCGCTCCAGCGCT-3' |  |
| *Entamoeba* spp. | 5'-GTTGATCCTGCCAGTATTATATG-3' | Verweij et al.2001 |
|  | 5'-CACTATTGGAGCTGGAATTAC-3' |  |
| *Trichuris* sp. | 5'-TGTAAATCTCCTGCCCAATGA-3' | Meekums et al.2015 |
|  | 5'-CGGTTTAAACTCAAATCACGTA-3' |  |
| *Ascaris* sp. | 5'-TATGAGCGTCATTTATTGGG-3' | Peng et al.2005 |
|  | 5'-GCATCACAATAGCCAACAAATAC-3' |  |
| *Isospora* spp. | 5'-GCATCCTCCTGGTGGCGCTT-3' | Ruttkowski et al.2001 |
|  | 5'-CGGCCATGCACCACC-3' |  |
| *Physaloptera* sp. | 5'-GTTAGGATATTGGGCAGGTT-3' | This study |
|  | 5'-CCGTATTCAAAGGAGGGT-3' |  |
| *Enterobius vermicularis* | 5'-CACTTGCTATACCAACAACAC-3' | Iniguez et al.2002 |
|  | 5'-GCGCTACTAAACCATAGACG-3' |  |
| *Strongyloides stercoralis* | 5'-TTCTAGTGTTGATTTGGCT-3' | Sharifdini et al.2015 |
|  | 5'-TTACCACCAAAACTAGGATC-3' |  |
| *Ancylostoma* spp. | 5'-CGTGCTAGTCTTCAGGACTTTG-3' | Palmer et al.,2007 |
|  | 5'-CGGGAATTGCTATAAGCAAGTGC-3' |  |
| *Balantidium coli* | 5'-GCTCCTACCGATACCGGGT-3' | Ponce-Gordo et al.2011 |
|  | 5'-GCGGGTCATCTTACTTGATTTC-3' |  |
| *Capillaria* spp. | 5'-GAAGCCTTAATAACTATTTCAGG-3' | Di Cesare et al.2012 |
|  | 5'-CCTGTTARRCCTCCRATACT-3' |  |
| *Oesophagostomum* sp. | 5'-TGTCGAACGATGCTTGCTTT-3' | Bott et al.2009 |
|  | 5'-TTAGTTTCTTTTCCTCCGCT-3' |  |
| *Trichostrongylus* sp. | 5'-TCGAATGGTCATTGTCAA-3' | Bott et al.2009 |
|  | 5'-TTAGTTTCTTTTCCTCCGCT-3' |  |
| *Cryptosporidium* spp. | 5'-AGCTCGTAGTTGGATTTCTG-3' | Khalil et al.2016 |
|  | 5'-TAAGGTGCTGAAGGAGTAAGG-3' |  |
| *Gongylonema pulchrum* | 5'-TTTGGGGCTCCTGAGGTTTATA-3'  5'-CAGAAATCTCTTCCATCACCTCGAT-3' | Liu et al.2015 |
| *Hafnia-*16S rRNA | 5'-AGAGTTTGATCCTGGCTCAG-3'  5'-GGTTACCTTGTTACGACTT-3' | Abbott et al., 2011 |
| *Hafnia-ampC* | 5'-GTTATCCGTGATTACCTGTCTGGC-3'  5'- CTGCCCCATATTGGCTTGCAC-3' | Jayol et al., 2017 |

^a^ represent codes for PCR detection of pathogens in General Administration of Quality Supervision, Inspection and Quarantine of China.

Supplementary Table 2. Antibiotic susceptibility tests of the *H. paralvei HN*

| Antibiotic | MIC (ug/mL) | SIR |
| --- | --- | --- |
| Amikacin | <=8 | S |
| Gentamicin | <=2 | S |
| Imipenem | <=1 | S |
| Meropenem | <=1 | S |
| Cefazolin | >16 | R |
| Ceftazidime | 2 | S |
| Cefotaxime | <=1 | S |
| Cefepime | <=2 | S |
| Aztreonam | <=2 | S |
| Ampicillin | 16 | R |
| Piperacillin | <=4 | S |
| Amoxicillin-Clavulanate | >16/8 | R |
| Ampicillin-Sulbactam | >16/9 | R |
| Piperacillin-Tazobactam | <=4/4 | S |
| Colistin | >2 | ND |
| Trimethoprim-Sulfamethoxazole | <=0.5/9.5 | S |
| Chloramphenicol | <=4 | S |
| Ciprofloxacin | <=0.5 | S |
| Levofloxacin | <=1 | S |
| Moxifloxacin | <=1 | ND |
| Tetracycline | <=2 | R |

Note: S, sensitivity; R, Resistance; ND, not date.
